# Effects of mineral waters on acid–base status in healthy adults: results of a randomized trial

**DOI:** 10.29219/fnr.v63.3515

**Published:** 2019-12-03

**Authors:** Paulina Wasserfurth, Inga Schneider, Alexander Ströhle, Josefine Nebl, Norman Bitterlich, Andreas Hahn

**Affiliations:** 1 Institute of Food Science and Human Nutrition, Leibniz University Hannover, Hannover, Germany; 2 Department of Biostatistics, Medicine and Service GmbH, Chemnitz, Germany

**Keywords:** mineral water, acid–base status, dietary acid load, net acid excretion

## Abstract

**Background:**

The ‘Western diet’ typically consumed in industrialized countries is characterized by high amounts of processed cereal grains and animal products while being low in vegetables, tubers, and fruits. This dietary behavior leads to imbalances of acid–base status in favor of the acids and may cause low-grade metabolic acidosis (LGMA) that is associated with negative effects on health in the long run, including urolithiasis, bone loss, and even cardiometabolic diseases. Therefore, it has become of great interest to find dietary strategies that can be used to neutralize the acid load associated with Western diets.

**Objective:**

The aim of this study was to investigate whether the diet-dependent net acid load can be reduced by the daily consumption of mineral waters with different bicarbonate content and different potential renal acid load (PRAL).

**Methods:**

A single-centered, randomized trial including 129 healthy men and women aged from 18 to 75 years was conducted. Participants consumed 1,500–2,000 mL of one of four mineral waters with different bicarbonate content and different PRAL values daily for 4 weeks: low bicarbonate, high PRAL (LBHP, HCO_3_^−^: 403.0 mg/L, PRAL: 10.7); medium-high bicarbonate, medium PRAL (MBMP, HCO_3_^−^ : 1816.0 mg/L, PRAL: −10.8); high bicarbonate, low PRAL (HBLP, HCO_3_^−^: 2451.0 mg/L, PRAL: −19.3); medium-high bicarbonate, low PRAL (MBLP, HCO_3_^−^: 1846.0 mg/L, PRAL: −22.1). Throughout the study, participants were asked to maintain their usual dietary habits. The primary outcome was the net acid excretion (NAE) measured in the 24-h urine output.

**Results:**

Consumption of the three mineral waters: MBMP, HBLP, and MBLP led to a significant decrease in NAE values. Within the MBMP group, the NAE could be reduced by 48% (*P* = 0.001), while consumption of HBLP led to a reduction of 68% (*P* < 0.001) and MBLP to a reduction of 53% (*P* = 0.001). Moreover, a slight increase in serum bicarbonate could also be observed in the groups that drank HBLP (*P* = 0.057) and MBLP (*P* = 0.001).

**Conclusion:**

Daily consumption of at least 1,500–2,000 mL of mineral water rich in bicarbonate (>1800.0 mg/L) with medium or low PRAL (<−11 mEq/L) can effectively reduce the NAE level by reducing the dietary acid load under free-living conditions in healthy adults.

## Popular scientific summary

- This was the first study to investigate the effect of mineral waters with varying mineralization on acid-base status in free-living, healthy adults.
- The results demonstrate that mineral waters rich in bicarbonate and with negative potential renal acid load (PRAL) can effectively reduce the net acid excretion (NAE), which reflects the dietary net acid load.
- Regular consumption of such mineral waters might be useful in preventing risks associated with a higher dietary acid load.

Since the beginning of the 20th century, it has been known that dietary composition affects acid–base homeostasis and urine pH in man (1, 2). Up to now, there is increasing evidence that even small diet-induced disturbances in acid–base status (low-grade metabolic acidosis; LGMA) may have negative effects on health in the long run, which may manifest as urolithiasis, bone loss and cardiometabolic diseases (3–5). On the other hand, it was shown that diets with high amounts of fruits and vegetables or alkaline supplements decreased calciuria and bone resorption markers in adults, underlining the importance of a well-balanced diet (6). However, the ‘Western diet’ typically consumed in industrialized countries is characterized by high amounts of processed cereal grains and red meat while being low in vegetables, tubers, and fruits. Meat and cereals are rich in the sulfur-containing amino acids methionine and cysteine whose oxidation to urea and carbon dioxide also yields sulfuric acid (7–9). The released protons add to the acid-pool in the blood, contributing to the diet-dependent net acid load. In contrast, base equivalents are formed through the metabolization of organic acid anions of alkali salts present in vegetables and fruits (10). However, the low consumption of base-producing foodstuffs cannot compensate the acidic load caused by an excessive consumption of animal products. As a consequence, most Western diets show net acid-producing effects of about 50–100 mEq per day (7, 11).

Against this background there is an increasing interest to find dietary strategies that can be used to neutralize the acid load of Western diets. In this context, the consumption of mineral waters rich in bicarbonate was suggested to be a suitable and practical source of alkali-formers (12). Moreover, Burckhardt stated that the consumption of mineral waters rich in bicarbonate is one of the most practical measures to increase the dietary alkali load (13). Based on the individual mineralization, the alkalinizing or acidifying effect of mineral water can be calculated as the potential renal acid load (PRAL) from its content of chloride (Cl^−^), sulfate (SO_4_^2−^) , potassium (K^+^), magnesium (Mg^2+^), calcium (Ca^2+^), and sodium (Na^+^) (14). While a positive PRAL indicates acidity, a negative PRAL indicates alkalinity.

Although Frassetto et al. and König et al. could demonstrate a positive effect of bicarbonate supplementation and alkaline supplements on blood and urinary parameters of acid base status, only one study by Heil examined the effect of an alkaline mineral water with a pH of 10 (15–17). Up to now, no data about the effect of bicarbonate-rich mineral waters on acid base status exists.

Therefore, we evaluated whether the regular consumption of mineral waters with different bicarbonate content and different PRAL can influence the acid–base status under non-standardized dietary conditions in omnivorous healthy adults.

## Materials and methods

### Study design

A single-center, randomized, controlled trial in parallel group design was conducted by trained professionals using standardized methods at the Institute of Food Science and Human Nutrition, Leibniz University Hannover, Germany. The study involved a screening phase, a 4-week run-in-phase and a 4-week intervention phase with 2 examination days; one at the beginning (*t*_0_) and one at the end (*t*_4_) of the intervention.

Ethical approval was provided by the Ethics Commission of the Medical Chamber of Lower Saxony (Hannover, Germany). In accordance with the guidelines of the Declaration of Helsinki, written informed consent was obtained from all subjects prior to their participation in the study. This study is registered in the German Clinical Trial Register (DRKS00012290).

### Subjects

Subjects were recruited via advertisements in local newspapers and public notice boards from the general population in Hannover, Germany. The main inclusion criteria for participation were: (A) age ≥ 18 and ≤ 75 years and (B) a body mass index (BMI) ≥ 18.5 and ≤32 kg/m^2^ (C) following an omnivorous diet.

Exclusion criteria were defined as: (A) intake of magnesium and/or calcium supplements; (B) intake of drugs that likely affect mineral absorption and/or acid–base status such as laxatives, proton pump inhibitors, diuretics, corticoids and isoflavonoids; (C) hormone replacement therapy 6 months before beginning of the study or during the intervention; (D) diagnosed and treated osteoporosis, urinary stones and intake of urine acidifying medicine; (E) suspicion and diagnosis of blood coagulation disorders, chronic gastrointestinal disorders (e.g. ulcers, Crohn’s disease, pancreatic insufficiency), cardiovascular diseases (angina pectoris, myocardial infarction, stroke, peripheral arterial occlusive disease, heart failure, cardiac arrhythmia), type 1 and 2 diabetes, renal insufficiency and liver diseases; (F) alcohol, drug and/or medicine dependency; (G) pregnancy or lactation; (H) retraction of the consent by the subject, concurrent participation in another clinical study and participation in a study in the last 30 days. Inclusion and exclusion criteria were audited using a structured questionnaire. Of the 175 people interested, 129 (74%) met the eligibility criteria and were randomly assigned by an outside researcher using block randomization to one of the four study groups.

### Test products and procedure

Four commercially available German mineral waters were tested during this study. The mineral waters were selected with regards to their bicarbonate content and their alkalizing or acidifying potential which is dependent on their different mineral content, defined by the respective PRAL. To evaluate the effect of different mineral waters, three mineral waters rich in bicarbonate (> 1,500 mg/L) and having various mineralizations were chosen. In contrast to that, one mineral water with low bicarbonate content and a rather acidic mineralization was chosen as well. With regards to those selection criteria the mineral waters were classified as follows: (1) LBHP, (2) MBMP, (3) HBLP (high bicarbonate low PRAL), and (4) MBLP. The composition of the test products is shown in Table 1. The LBHP mineral water had the lowest bicarbonate content of 403.0 mg/L while HBLP had the highest of 2,451 mg/L. Concentrations of bicarbonate in MBMP and MBLP were almost identical (1,816 mg/L and 1,846 mg/L respectively). MBMP (PRAL = −10.8), HBLP (PRAL = −19.3), and MBLP (PRAL = −22.1) showed negative PRAL-values indicating alkalinity while LBHP (PRAL = 10.7) had a positive value indicating acidity. The PRAL index for each individual mineral water was calculated as described by Wynn et al. (14). To evaluate the long-term effect of the mineral waters on the acid–base status, participants were advised to drink 1,500–2,000 mL of the respective mineral water daily over a period of 4 weeks. Additional fluid requirements were allowed to be covered with tap water, tea, coffee, juices, and soft drinks. The amount participants were advised to drink was based on the recommendations of the nutrition societies of Germany, Austria, and Switzerland (18). The compliance regarding the daily mineral water consumption was assessed with a questionnaire at the second examination. Participants were assessed as compliant when they reported a daily intake of 1,500–2,000 mL of the respective mineral water.

**Table 1:** Composition of the four tested mineral waters per liter

	LBHP	MBMP	HBLP	MBLP
HCO_3_^−^ (mg/L)	403.0	1816.0	2451.0	1846.0
HCO_3_^−^ (mmol/L)	6.6	29.8	40.2	30.3
Ca^2+^ (mg/L)	528.0	348.0	168.0	98.7
Mg^2+^ (mg/L)	124.0	108.0	241.0	59.2
Na^+^ (mg/L)	28.8	118	261	564
K^+^ (mg/L)	6.9	10.5	37.4	16.1
Cl^−^ (mg/L)	28.9	39.7	14.0	139
SO_4_^2−^ (mg/L)	1463.0	38.3	17.0	39.0
PO_4_^3−^ (mg/L)	−*	0.1	1.1	−*
PRAL (mEq/L)	10.7	−10.8	−19.3	−22.1

* Phosphate content below the analytical limit of determination.

The participants consumed 37.5 μg of Vitamin D (Taxofit^®^ Vitamin D_3_ 1,500 I.E. Depot Tabletten, MCM Klosterfrau Vertriebsgesellschaft mbH, Cologne, Germany) daily for4 weeks before and during the study to ensure adequate vitamin D status. For the duration of the study, participants were asked to maintain their usual dietary behavior.

### Dietary records and calculation of PRAL and NEAP

To assess dietary habits, participants completed 3-day dietary records at the beginning and the end of the study. Participants were instructed to log their food over 3 consecutive days, including 2 week days and 1 weekend day. The records were checked by nutritionists for completeness, readability, and plausibility. Ambiguities were clarified with subjects, if necessary. Nutrient intakes were estimated from the participant’s3-day dietary record using the PRODI6.4^®^ software (Nutri-Science GmbH, Freiburg, Germany). The PRAL of the food consumed by the participants was calculated using the formula proposed by Remer and Manz (PRAL_1995_) and Remer et al. (PRAL_2003_) (19, 20). As the formula by Remer_2003_ omits dietary sodium and chloride intakes, both calculation models were used. Moreover, the net endogenous acid production (NEAP_1995_ and NEAP_2003_) was determined according to both formulae as well. The detailed algorithmic formulae for the calculations of the two NEAP models are as follows (11):

NEAP_1995/2003_ (mEq/d) = PRAL_1995/2003_ (mEq/d) + OA_est_ (mEq/d), where OA_est_ represents anthropometry-based estimated urinary organic anions. The computation of OA_est_ was carried out by using the following formula: OA_est_ (mEq/d) = 0.007184×height (cm)^0.725^×weight (kg)^0.425^×41/1.73

### Urine sampling and biochemical indices measurement

For collection of 24-h urine samples, all participants received personal and written instructions as well as preservative-free plastic containers (Sarstedt AG& Co. KG, Nümbrecht, Germany). Subjects were asked to start the collection the day before their examination, after disposing of the initial morning urine and collecting until the following day (including the morning urine). Immediately after delivery, the urine was mixed thoroughly, aliquoted, and stored at −22°C till analysis (21). The urine following the initial morning urine was collected as spot urine after arrival of the subjects at the research institute. Urine pH was measured using a pH meter (Mettler-Toledo, Gießen, Germany). Titratable acids (TA), ammonium (NH_4_^+^), and HCO_3_^−^ were measured according to the method stated by Lüthy et al. (22). Because excretion of HCO_3_^−^ is negligible at a urine pH of 6.2 or below, HCO_3_^−^values of the concerned sample were set to zero.

Further urinary analyses were performed at the Hannover Medical Care Center of the LADR network.

### Blood sampling and biochemical indices measurement

After arrival at the research institute and delivery of the urine, fasting venous blood was drawn from each participant after a 12-h fast using EDTA and serum tubes (Sarstedt AG& Co. KG, Nümbrecht, Germany). During the fast, participants were allowed to drink tap water if needed.

Blood pH, partial pressure of carbon dioxide (pCO_2_), base excess (BE) and bicarbonate (HCO_3_^−^) were analyzed using the epoc^®^ Blood Analysis System (Siemens Healthcare GmbH, Erlangen, Germany). Further analyses were performed at the Hannover Medical Care Center of the LADR network.

### Statistical analysis

Data are presented as mean ± standard deviation (SD). Distribution of data was assesed with Shapiro-Wilk test. Differences in baseline characteristics were compared using Kruskal-Wallis test for continuous variables and Fisher’s exact test for nominal variables. To compare differences before and after the intervention within group differences were analyzed with Wilcoxon test while differences between groups were analyzed using Kruskal-Wallis test. If differences were statistically significant a Bonferroni post-hoc analysis was performed. Values of p < 0.05 were regarded as statistically significant. All statistical analyses were carried out using the SPSS Software (Version 23.0; SPSS Inc., Chicago, IL, USA).

## Results

### Study population

**Fig. 1:**
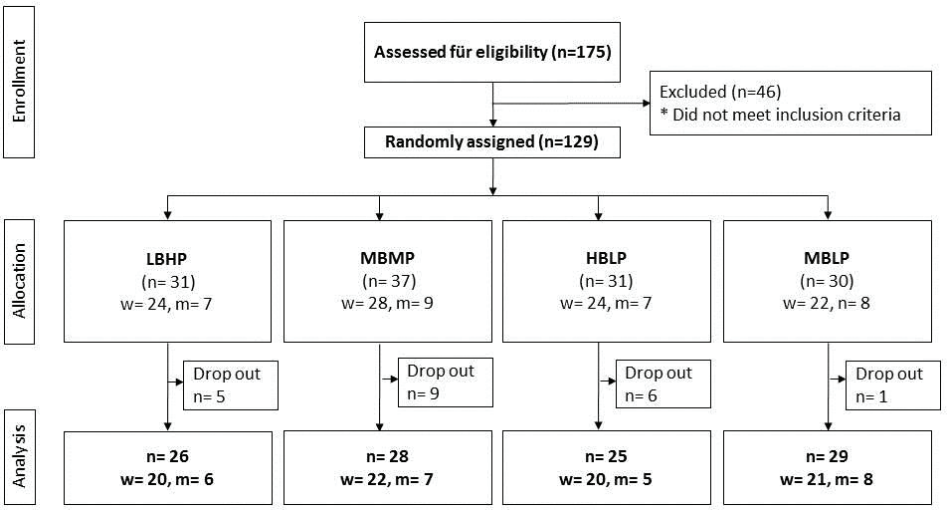
Flow diagram of participants in the trial.

Of the 129 participants included in this study, 21 discontinued the study because of different reasons such as pregnancy, illness, or other personal reasons. The characteristics of the study population at baseline are shown in Table 2. Between the four study groups, there were no significant differences in sex, age, hip and waist circumference, height, weight, BMI, blood pressure, and pulse frequency. Altogether a total of 108 subjects (women: *n* = 83 (76%), men: *n* = 26 (24%) completed the study (Fig. 1).

**Table 2:** Baseline characteristics of the study population

	LBLP	MBMP	HBHP	MBHP	*P*
*n*	31	37	31	30
Sex (f/m)	(24 / 7)	(28 / 9)	(24 / 7)	(22 / 7)	0.984^a^
Age (years)	42.3 ± 16.8	43.6 ± 15.9	43.5 ± 16.1	43.7 ± 16.4	0.967^b^
Hip circumference (cm)	77.9 ± 8.29	79.3 ± 10.5	77.1 ± 9.53	81.1 ± 15.1	0.807^b^
Waist circumference (cm)	98.1 ± 9.33	100 ± 9.68	96.4 ± 9.00	98.9 ± 10.6	0.606^b^
Height (cm)	169 ± 8.6	170 ± 11.0	170 ± 7.4	170 ± 7.8	0.954^b^
Body weight (kg)	69.4 ± 11.35	73.1 ± 16.9	70.5 ± 12.1	72.6 ± 13.9	0.839^b^
BMI (kg/m^2^)	23.8 ± 2.7	23.6 ± 2.5	23.6 ± 2.5	23.6 ± 2.7	0.826^b^
SBP (mmHg)	128 ± 17.7	122 ± 13.5	129 ± 14.3	127 ± 18.3	0.460^b^
DBP (mmHg)	72.9 ± 11.0	71.7 ± 8.8	77.3 ± 6.9	71.8 ± 10.9	0.089^b^

*Note:* f, females; m, males; BMI, body-mass index; SBP, systolic blood pressure; DBP, diastolic blood pressure.

a Exact Fisher test.

b Kruskal–Wallis test.

### Dietary intake of PRAL-related nutrients and dietary acid load

The intakes of energy, macronutrients, and PRAL-related nutrients of the four subgroups at baseline (*t*_0_) and after the intervention (*t*_4_) are shown in Table 3. Although the HBLP group showed a significantly lower energy intake at *t*_4_(*P* = 0.016), overall, no intergroup differences could be detected either at *t*_0_ or at *t*_4_.

**Table 3:** Dietary intake of macronutrients, electrolytes, and dietary acid load at baseline (*t*_0_) and at 4 weeks (*t*_4_)

	LBHP			MBMP			HBLP			MBLP			Intergroup comparison	
	*t*_0_	*t*_4_	*P*^a^	*t*_0_	*t*_4_	*P*^a^	*t*_0_	*t*_4_	*P*^a^	*t*_0_	*t*_4_	*P*^a^	*P*^b^*t*_0_	*P*^b^*t*_4_
Energy intake (kJ)	9,700 ± 2,420	8,830 ± 2,060	0.074	8,810 ± 2,200	9,230 ± 2,110	0.408	9,850 ± 2,990	8,650 ± 2,070	0.016	8,860 ± 2,410	9,080 ± 2,770	0.831	0.115	0.534
Protein (g)	88.3 ± 30.7	84.2 ± 25.5	0.491	82.2 ± 26.1	88.4 ± 23.3	0.173	85.4 ± 26.8	75.8 ± 21.9	0.077	76.5 ± 26.0	80.6 ± 26.1	0.221	0.351	0.272
Ca^2+^ (mg)	851 ± 239	863 ± 253	0.895	822 ± 376	783 ± 381	0.842	790± 234	773 ± 225	0.438	778 ± 329	756 ± 269	0.761	0.475	0.116
Mg^2+^ (mg)	356 ± 92.0	302 ± 88.3	**0.005**	351 ± 111	353 ± 112	0.822	362 ± 136	286 ± 80.9	**0.001**	312 ± 101	310 ± 106	0.808	0.701	0.096
K^+^(g)	3.33 ± 0.79	2.95 ± 0.90	**0.020**	3.26 ± 0.91	3.25 ± 0.97	0.960	3.13 ± 0.66	2.79 ± 0.64	**0.026**	2.91 ± 0.82	2.84 ± 0.88	0.349	0.570	0.341
PO_4_^3−^(g)	1.48 ± 0.31	1.36 ± 0.31	0.191	1.36 ± 0.43	1.47 ± 0.39	0.217	1.40 ± 0.39	1.27 ± 0.31	0.066	1.25 ± 0.42	1.30 ± 0.44	0.371	0.148	0.241
Na^+^(mg)	2.33 ± 0.92	2.36 ± 0.94	0.791	1.98 ± 0.82	2.67 ± 1.07	**0.004**	2.31 ± 0.93	2.50 ± 1.85	0.567	2.93 ± 3.79	2.50 ± 1.12	0.393	0.100	0.351
Cl^−^ (mg)	2.83 ± 0.93	2.93 ± 1.12	0.874	2.89 ± 1.00	3.83 ± 1.82	**0.043**	3.03 ± 1.24	3.30 ± 2.24	0.814	4.20 ± 5.59	3.61 ± 1.47	0.191	0.913	0.062
PRAL_1995_ (mEq/d)	−12.1 ± 32.1	−7.78 ± 26.5	0.426	−1.58 ± 24.5	3.08 ± 19.7	0.303	−5.19 ± 24.6	−6.05 ± 26.8	0.598	−3.08 ± 20.7	4.41 ± 14.6	**0.008**	0.538	0.152
PRAL_2003_ (mEq/d)	7.73 ± 25.4	10.7 ± 18.2	0.508	2.30 ± 18.2	10.2 ± 15.1	0.075	8.19 ± 22.0	8.12 ± 16.6	0.779	4.58 ± 17.0	10.1 ± 15.4	0.237	0.909	0.895
NEAP_1995_ (mEq/d)	30.1 ± 32.3	34.9 ± 27.3	0.437	42.4 ± 25.7	47.0 ± 23.3	0.315	37.8 ± 25.2	36.9 ± 27.3	0.598	40.3 ± 22.8	47.3 ± 16.7	**0.011**	0.351	0.161
NEAP_2003_(mEq/d)	49.9 ± 26.4	53.4 ± 20.2	0.515	46.3 ± 20.0	54.2 ± 18.8	0.080	51.2 ± 18.3	51.1 ± 18.3	0.779	48.0 ± 18.7	53.1 ± 17.5	0.289	0.990	0.882

All *p* values < 0.05 were accounted as statistically significant.

a Wilcoxon test.

b Kruskal–Wallis test.

Further, at baseline, no significant differences in intake of protein, Ca^2+^ and phosphorus (PO_4_^3−^) could be detected between the four groups. However, the intake of Mg^2+^ differed significantly between the two examinations within the groups LBHP (*P* = 0.005) and HBLP (*P* = 0.001). In both groups, there was a lower intake of Mg^2+^ at *t*_4_. The same could be observed for potassium (K^+^) intake. Again, LBHP (*P* = 0.020) and HBLP (*P* = 0.026) showed a lower intake at *t*_4_. With regards to Na^+^ and Cl^−^ intake, a significantly higher intake could be observed in the MBMP group at *t*_4_.

Although there were differences in Mg^2+^, K^+^, Na^+^ und Cl^−^intake, no differences in PRAL_2003_and NEAP_2003_ could be detected within all study groups at *t*_0_ and *t*_4_ (Table 3). However, with regards to PRAL_1995_ and NEAP_1995_ that took Na^+^ and Cl^−^ into account, a significant increase from *t*_0_ to *t*_4_ could be observed in the MBLP group (*P* = 0.008 and *P* = 0.011 respectively). When compared amongst each other, no intergroup differences could be detected for any of the two examinations.

### Mineral intake through mineral water consumption

Through daily consumption of at least 1,500 mL of one of the four mineral waters participants had an additional mineral intake, which can be derived from Table 1. The highest additional Ca^2+^intake of at least 792 mg was achieved in the group that drank the LBHP water while the lowest intake of 148 mg was in the MBLP group. Mg^2+^intake was highest in the HBLP group (362 mg) and lowest in the MBLP group (88.8 mg). However, the MBLP group had the highest Na^+^ (846 mg) intake. SO_4_^2−^intake was highest in the LBHP group (2,195 mg) and lowest in the HBLP group (25.5 mg). Due to the low amounts of K^+^, Cl^−^, and PO_4_^3−^, the additional intake of these minerals was considered as not relevant.

### Effects of mineral waters on venous parameters of the acid–base status

As shown in Table 4, there was a significant reduction of blood pH within LBHP (*P* = 0.003) and MBMP (*P* = 0.006) between baseline and *t*_4_, whereas no significant change was noted for HBLP and MBLP. With regards to HCO_3_^−^, for MBLP, a significant increase was detected (*P* = 0.001) whereas for HBLP significance was marginally missed (*P* = 0.057). Moreover, with MBLP, pCO_2_ (*P* = 0.002) and BE (*P* = 0.003) increased, while pO_2_ (*P* = 0.007) decreased significantly in this group as well. The intergroup comparison revealed a significant difference among the groups regarding the blood pH at *t*_4_ (*P* = 0.041).

**Table 4:** Effects of mineral waters on the venous parameters of the acid–base status

	LBHP			MBMP			HBLP			MBLP			Intergroup comparison	
	*t*_0_	*t*_4_	*P*^a^	*t*_0_	*t*_4_	*P*^a^	*t*_0_	*t*_4_	*P*^a^	*t*_0_	*t*_4_	*P*^a^	*P*^b^*t*_0_	*P*^b^*t*_4_
Blood pH	7.37 ± 0.03	7.36 ± 0.03	**0.003**	7.37 ± 0.02	7.36 ± 0.02	**0.006**	7.38 ± 0.03	7.37 ± 0.03	0.358	7.37 ± 0.03	7.36 ± 0.03	0.142	0.673	**0.041**
HCO_3_^−^ (mmol/L)	26.6 ± 2.35	26.9 ± 2.52	0.397	27.2 ± 1.55	27.5 ± 2.38	0.976	26.7 ± 1.75	27.5 ± 2.45	0.057	25.9 ± 2.58	27.3 ± 2.57	**0.001**	0.203	0.746
pCO_2_ (mmHg)	45.7 ± 5.63	48.2 ± 6.02	0.040	47.5 ± 4.81	49.4 ± 5.94	0.153	45.6 ± 4.82	47.5 ± 5.73	0.089	44.7 ± 6.51	48.2 ± 6.18	**0.002**	0.240	0.612
pO_2_ (mmHg)	22.4 ± 14.1	29.7 ± 21.1	0.155	29.7 ± 10.8	25.2 ± 11.9	0.143	29.3 ± 12.7	27.0 ± 14.4	0.377	36.2 ± 22.2	27.1 ± 14.0	**0.007**	0.428	0.695
BE (mmol/L)	0.82 ± 1.71	0.61 ± 1.89	0.518	1.25 ± 1.03	1.16 ± 1.70	0.558	0.97 ± 1.34	1.56 ± 1.80	0.056	0.28 ± 1.81	1.07 ± 1.80	**0.003**	0.124	0.289

All *p* values < 0.05 were accounted as statistically significant.

a Wilcoxon test.

b Kruskal–Wallis test.

### Effects of mineral waters on electrolytes and urine parameters of the acid–base status

Results from the urinary analysis are presented in Table 5. Consumption of LBHP led to a significant decrease (*P* = 0.003) of the pH of spot urine, whereas consumption of the bicarbonate rich mineral waters led to an increase. For MBMP and MBLP, those changes were significant (*P* = 0.007; *P* = 0.025). A similar result could be observed for pH measured in 24-h urine. Again, LBHP decreased urinary pH, while the other study groups showed an increase. Increases were significant in HBLP (*P* ≤ 0.001) and MBLP (*P* = 0.008). Although the pH of the spot-urine and 24-h urine did not differ at baseline, both parameters showed a significant difference among all study groups after 4 weeks (*P* ≤ 0.001).

**Table 5:** Effects of mineral waters on urine electrolytes and urine parameters of the acid–base status

	LBHP			MBMP			HBLP			MBLP			Intergroup comparison	
	*t*_0_	*t*_4_	*P*^a^	*t*_0_	*t*_4_	*P*^a^	*t*_0_	*t*_4_	*P*^a^	*t*_0_	*t*_4_	*P*^a^	*P*^b^*t*_0_	*P*^b^*t*_4_
Spot urine pH	5.99 ± 0.58	5.54 ± 0.63	**0.003**	5.86 ± 0.59	6.25 ± 0.75	**0.007**	6.16 ± 0.71	6.33 ± 0.49	0.252	5.85 ± 0.72	6.13 ± 0.62	**0.025**	0.277	**<0.001**
Urine volume (L/24 h)	2.33 ± 1.08	2.71 ± 1.11	0.072	2.45 ± 1.10	2.86 ± 1.01	0.059	2.18 ± 0.77	2.58 ± 0.74	**0.013**	1.89 ± 0.88	2.49 ± 0.73	**<0.001**	0.145	0.639
Urine pH (24 h)	6.23 ± 0.66	6.08 ± 0.65	0.143	6.18 ± 0.67	6.48 ± 0.49	0.068	6.11 ± 0.69	6.79 ± 0.42	**<0.001**	6.24 ± 0.78	6.73 ± 0.70	**0.008**	0.887	**<0.001**
TA (mmol/L)	5.70 ± 7.63	3.59 ± 4.05	0.068	4.87 ± 7.83	2.23 ± 3.24	**0.041**	6.25 ± 10.3	2.38 ± 3.23	**<0.001**	6.84 ± 11.7	2.98 ± 4.50	**0.014**	0.605	0.699
NH_4_^+^(mmol/L)	30.4 ± 15.1	25.1 ± 12.7	0.148	24.4 ± 9.74	18.4 ± 4.98	**0.003**	30.0 ± 19.2	19.9 ± 11.0	**<0.001**	32.4 ± 23.9	18.6 ± 6.72	**<0.001**	0.606	0.052
HCO_3_^−^ (mmol/L)	7.01 ± 10.66	4.42 ± 6.57	0.216	4.57 ± 5.31	7.91 ± 4.91	**0.007**	6.49 ± 11.91	12.92 ± 7.71	**0.002**	8.09 ± 15.43	9.18 ± 6.41	0.175	0.990	**<0.001**
Creatinine (mmol/L)	1.25 ± 0.30	1.18 ± 0.33	0.224	1.27 ± 0.48	1.31 ± 0.45	0.846	1.45 ± 0.41	1.42 ± 0.51	0.292	1.19 ± 0.50	1.27 ± 0.45	0.248	0.104	0.289
Uric acid (g/dL)	0.53 ± 0.13	0.51 ± 0.17	0.310	0.51 ± 0.19	0.53 ± 0.21	0.638	0.57 ± 0.19	0.57 ± 0.25	0.766	0.45 ± 0.19	0.49 ± 0.15	0.156	0.114	0.574
Ca^2+^(mmol/L)	4.17 ± 1.86	6.23 ± 2.53	**<0.001**	4.52 ± 2.08	5.41 ± 2.26	**0.010**	3.98 ± 2.16	4.66 ± 2.13	**0.022**	4.02 ± 2.19	4.65 ± 2.48	0.085	0.654	**0.037**
Mg^2+^ (mmol/L)	4.17 ± 1.20	5.58 ± 2.10	**0.001**	4.23 ± 2.03	5.46 ± 1.45	**0.003**	4.41 ± 1.85	6.67 ± 1.73	**<0.001**	3.59 ± 1.68	4.34 ± 1.88	**0.025**	0.248	**<0.001**
K^+^ (mmol/L)	68.9 ± 22.5	68.2 ± 23.9	0.746	68.6 ± 31.0	73.4 ± 33.7	0.586	74.0 ± 23.9	79.0 ± 28.4	0.257	55.3 ± 25.9	59.8 ± 25.0	0.357	0.092	**0.020**
NAE (mEq/L)	29.29 ± 15.85	24.18 ± 11.60	0.339	25.77 ± 17.92	13.38 ± 9.04	**0.001**	34.50 ± 26.24	11.22 ± 10.99	**<0.001**	32.44 ± 26.34	15.31 ± 13.54	**0.001**	0.507	**<0.001**

*Note*: TA, titrable acid.

All *p* values < 0.05 were accounted as statistically significant.

a Wilcoxon test.

b Kruskal–Wallis test.

Titrimetric analysis of 24-h urine revealed a significant decline in TA and NH_4_^+^ within groups’ MBMP (TA: *P* = 0.041; NH4: *P* = 0.003), HBLP (TA: *P* ≤ 0.001; NH_4_^+^: *P* ≤ 0.001), or MBLP (TA: *P* = 0.014; NH_4_^+^: *P* ≤ 0.001). With regard to concentrations of HCO_3_^−^, a significant increase could be observed in MBMP (*P* = 0.007) and HBLP (*P* = 0.002). The intergroup comparison also revealed a significant difference in HCO_3_^−^ excretion at *t*_4_ (*P* ≤ 0.001).

**Fig. 2:**
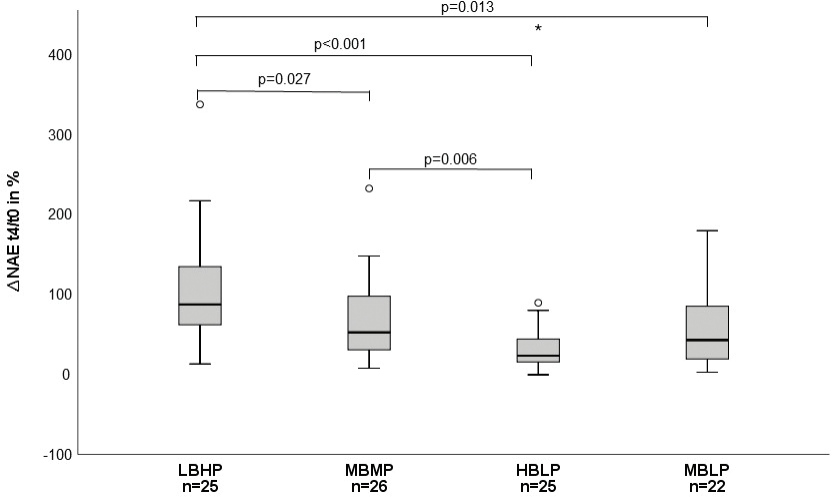
Percentage change of the net acid excretion (NAE) from *t*_0_ to *t*_4_.

Drinking of at least 1,500 mL of MBMP, HBLP, and MBLP led to a distinct reduction of the net acid excretion (NAE) when compared within the groups (MBMP: *P* = 0.001, HBLP: *P* ≤ 0.001, MBLP: *P* ≤ 0.001) and also when compared among the groups at *t*_4_ (*P* ≤ 0.001). The relative change in NAE from the baseline to *t*_4_ is shown in Fig. 2.

## Discussion

To the best of our knowledge, this was the first study to investigate the effect of mineral waters with different bicarbonate content and different PRAL on the acid–base status in healthy adults, under free-living conditions. Our findings show that daily consumption of 1,500–2,000 mL alkaline mineral water with a bicarbonate content of >29.7 mmol/L can effectively reduce the dietary acid load and therefore positively influence the acid–base status.

### Influence on venous acid–base parameters

In general, the acid–base status can be determined from the blood pH, which is kept in a narrow range of 7.36–7.44 by a number of buffer systems, the lungs, and the kidneys (23). Disturbances of the acid–base-balance are characterized by a pH of <7.36 (acidosis) or >7.44 (alkalosis) and can be either due to respiratory or metabolic disorders (24, 25). To identify whether a disturbance is of respiratory or metabolic nature, not only blood pH but also the pCO_2_ as a marker for respiratory disorders, and the concentration of bicarbonate as well as BE, as a marker for metabolic disorders, should be measured (26–29).

A previous study conducted by Heil has shown that consumption of a mineral-rich alkaline mineral water with a pH of 10 can lead to an increase in blood pH (16). Moreover, König et al. demonstrated that a supplement rich in alkaline minerals can not only increase blood pH but also the blood HCO_3_^−^concentration, BE, and pCO_2_ (17). In contrast to those studies, the present study examined the effect of not only one, but compared four mineral waters with different PRAL values and different bicarbonate content. The alkalinity of the mineral waters was defined by the respective PRAL. With regards to the findings from the studies earlier described, we hypothesized that the daily consumption of the three mineral waters with negative PRAL would positively influence venous parameters of acid–base metabolism, whereas consumption of the mineral water with a positive PRAL might have a negative effect. Nevertheless, no favorable effects of any of the alkaline mineral waters could be observed in terms of blood pH. Quite in contrast, all study groups showed a decrease in blood pH, with a significant reduction in the groups that drank LBHP and MBHP. Although those results differ from previous findings, it has to be pointed out that the observed changes in blood pH were minimal, and still within the reference range and therefore physiologically irrelevant. In addition, blood pH values of all participants were within the reference range at the beginning as well as at the end of the study. Furthermore, it has to be noted that the present study investigated the long-term effect of mineral water consumption over a period of 4 weeks with measurements at the beginning and at the end of the study. In contrast, Heil et al. investigate rather short-term effects over 3 h or 2 weeks and measured blood pH multiple times throughout their studies (16, 17). In our case, it is uncertain if and how blood pH fluctuated throughout the study, for example it rose directly after consumption of the alkaline waters. However, it has to be noted that the blood pH levels reported in this study are generally lower when compared to other studies. This can be explained by the fact that we used venous blood for the blood gas analyses and not arterial blood as the other studies described did. In any case, Awasthi et al. showed that venous blood gas analysis is a suitable alternative to arterial blood gas analysis but will yield a lower, more acidic, blood pH value (30).

With regards to blood HCO_3_^−^ concentration, BE and pCO_2_, the present study could show similar findings as described by König et al. within the group that drank the MBLP mineral water (17). As this mineral water was the one with the second highest bicarbonate content, those results may indicate that alkalinity might be more important than bicarbonate content with regards to metabolically influencing venous parameters of the acid–base metabolism.

### Influence on urinary acid–base parameters

Urinary parameters of acid–base homeostasis are particularly regulated by the kidneys, which control the acid–base homeostasis by excretion of excess acids and reabsorption of filtrated bicarbonate. In particular, excess acids can be eliminated via urine in three different forms: (1) as free hydrogen ions; (2) as TA, meaning that hydrogen ions are bound to buffering substances such as phosphate, sulfate, or organic acids and (3) as ammonia (31).

In evaluating the acid–base status, urinary pH was found to be a valid indicator and that the pH measured in 24-h urine also reflects the NAE (32, 33). Because earlier studies have shown that mineral waters rich in bicarbonate can lead to an increase in urinary pH, we expected to find similar results in those groups that drank the three mineral waters with highest bicarbonate contents and alkaline PRAL (34, 35). On the contrary, one could assume that consumption of the mineral water with low bicarbonate content and positive PRAL may lead to a decrease in the pH. With regard to pH values measured in spot urine, those expectations were met; however, the alteration in the HBLP group was not significant. In any case, it has to be noted that this group already had the highest urinary pH at baseline. Similar results were also found for the pH measured in 24-h urine, though these alterations were not significant in the HBLP and MBLP group. Altogether, alkalinity seemed to play a more prominent role in influencing the urinary pH than bicarbonate content.

In any case, the dietary acidic load can reliably be measured in 24-h urine by analyzing the NAE. Frassetto et al. showed that supplementation with 60–120 mmol potassium bicarbonate per day for 18 days led to a significant decrease of NAE in postmenopausal women when consuming a standardized diet (15). The present study could support those findings by showing that consumption of at least 1,500 mL of mineral water containing a minimum of ~45 mmol bicarbonate (MBMP, HBLP, MBLP) led to a significant reduction of the NAE when dietary habits remain stable.

However, it has to be noted that with regard to the dietary records of the participants, the NAE was lower than expected at baseline. One reason for the lower NAE may be an overestimation of HCO_3_^−^ occurring through the titration method proposed by Lüthy et al. (22). Actually, for physico-chemical reasons, HCO_3_^−^ excretion is negligible at pH values less than or equal to 6.2. Therefore, we have calculated the NAE by setting HCO_3_^−^ values at a pH of ≤ 6.2–0. Interestingly, a study by Ausman et al. showed that a urinary pH of ~6.18, which is comparable to the pH values obtained from our participants, showed a predicted NAE of <20 mEq/d (36).

Against this background, it has to be noted that although we only included omnivorous participants in our study, we assume that our study sample of healthy volunteers were more health conscious than the general population, which could be reflected in the lower NAE values.

### Mineral water intake and mineral status

Depending on their individual mineralization level, consumption of mineral waters can also positively influence the overall mineral balance, particularly with regards to calcium and magnesium. According to the second German National Nutrition Survey (NVS II), 46.1% of all males and 55.2% of all females did not meet the current intake recommendation for calcium. Moreover, an insufficient magnesium intake could be observed in 26.1% of all males and 28.5% of all females (37). Throughout this study, all study groups showed an average dietary calcium intake of 777.5–850.6 mg/d at *t*_0_and 755.5–863.3 mg/d at *t*_12_, which is also below the dietary reference value of the nutrition societies of Germany, Austria, and Switzerland (1,000 mg/d for men and women aged 19–65 years and older) (18). However, by consumption of 1,500 mL of the respective test product, the LBHP (792 mg/1.5 L), MBMP (522.0 mg/1.5 L), and HBLP (252.0 mg/L) groups could meet or even exceed the dietary reference value. With regards to dietary magnesium intake, almost all study groups could meet the dietary reference value (350 mg/d for adult males, 300 mg/d for adult females), showing an average intake of 311.7–361.7 mg/d at *t*_0_ and of 288.6–353.3 mg/d at *t*_12_ (18). Nonetheless, bioavailability of minerals from foodstuff can be dependent on other compounds, such as phytic acid which lowers bioavailability of minerals like magnesium and calcium from whole meal bread (38). Interestingly, studies could already show that magnesium and calcium bioavailability from different mineral waters is comparable to bioavailability from dietary supplements or foodstuff, making mineral water consumption a practical measure to increase mineral intake (39, 40). Moreover, an additional sulfate intake as seen in the LBHP group was shown to have beneficial effects on the gastrointestinal tract in patients with functional constipation (41).

### Strengths and limitations of the study

Strengths of the study included the testing of not only one, but multiple mineral waters with different mineralization, for example different calcium, magnesium, and sulfate contents as well as different bicarbonate contents. Moreover, the effect of the mineral waters was measured over a comparatively long intervention period, that is, 4 weeks. Other previous studies investigated rater short-term effects over a few hours or 2 weeks (16, 17). Lastly, the free-living conditions of the participants allow the direct transfer of knowledge gained from this study to everyday life. One limitation of the study was the monitoring of the participants’ dietary behavior. Although 3-day dietary records are a commonly used tool to document the dietary intake, they also have limitations as they rely on self-reported data. This means the data may potentially biased by for example wrong estimation of portion sizes, memory of the subjects, or incomplete reporting (42). With regards to portion sizes, self-reported weighted food records may yield more accurate estimation. Moreover, the database of the nutrition software used for analyses of the dietary records was lacking some of the foods consumed by the participants, implying that they had to be substituted with foods available in the database. This however, can lead to small inaccuracies regarding micro- and macronutrient intake of the participants. Additionally, as already stated, it has to be noted that the dietary behavior of the study participants was well balanced, which is also reflected by the already low NAE at baseline. Another limitation of our study was the audition of the participant’s compliance, which was conducted solely via questionnaires at the end of the study. However, this method seemed to be the best choice to keep the free-living conditions during the intervention and to avoid organizational issues.

## Conclusion

Taken together, this study could show that alkaline mineral waters rich in bicarbonate (>1818.0 mg/L) and with a medium or low PRAL (<−10.8) can effectively reduce the NAE by reducing the dietary acid load under free-living conditions in healthy adults. Therefore, regular consumption of alkaline bicarbonate rich mineral waters might be useful in preventing risks associated with a higher dietary acid load as can typically be found in Western diets. Moreover, mineral waters that are not only rich in bicarbonate but also in other minerals, such as magnesium and calcium, can improve mineral supply.

## References

[1] Blatherwick NR The specific role of foods in relation to the composition of urine. Arch Intern Med 1914; XIV: 409. doi: 10.1001/archinte.1914.00070150122008.

[2] Sherman HC, Gettler AO The balance of acid-forming and base-forming elements in foods, and its relation to ammonia metabolism. J Biol Chem 1912; 11: 323–38. doi: 10.1136/bmjopen-2015-010438

[3] Della Guardia L, Roggi C, Cena H Diet-induced acidosis and alkali supplementation. Int J Food Sci Nutr 2016; 67: 754–61. doi: 10.1080/09637486.2016.1198889. 27338594

[4] Jayedi A, Shab-Bidar S Dietary acid load and risk of type 2 diabetes: a systematic review and dose–response meta-analysis of prospective observational studies. Clin Nutr ESPEN 2018; 23: 10–18. doi: 10.1016/j.clnesp.2017.12.005. 29460782

[5] Souto G, Donapetry C, Calviño J, Adeva MM Metabolic acidosis-induced insulin resistance and cardiovascular risk. Metab Syndr Relat Disord 2011; 9: 247–53. doi: 10.1089/met.2010.0108. 21352078 PMC3155690

[6] Lambert H, Frassetto L, Moore JB, Torgerson D, Gannon R, Burckhardt P, et al. The effect of supplementation with alkaline potassium salts on bone metabolism: a meta-analysis. Osteoporos Int 2015; 26: 1311–18. doi: 10.1007/s00198-014-3006-9. 25572045

[7] Adeva MM, Souto G Diet-induced metabolic acidosis. Clin Nutr 2011; 30: 416–21. doi: 10.1016/j.clnu.2011.03.008. 21481501

[8] Cordain L, Eaton SB, Sebastian A, Mann N, Lindeberg S, Watkins BA, et al. Origins and evolution of the Western diet: health implications for the 21st century. Am J Clin Nutr 2005; 81: 341–54. doi: 10.1093/ajcn.81.2.341. 15699220

[9] Pizzorno J, Frassetto LA, Katzinger J Diet-induced acidosis: is it real and clinically relevant? Br J Nutr 2010; 103: 1185–94. doi: 10.1017/S0007114509993047. 20003625

[10] Remer T Influence of nutrition on acid-base balance – metabolic aspects. Eur J Nutr 2001; 40: 214–20. doi:10.1007/s394-001-8348-1. 11842946

[11] Ströhle A, Waldmann A, Koschizke J, Leitzmann C, Hahn A Diet-dependent net endogenous acid load of vegan diets in relation to food groups and bone health-related nutrients: results from the German Vegan Study. Ann Nutr Metab 2011; 59: 117–26. doi: 10.1159/000331572. 22142775

[12] Rylander R Drinking water constituents and disease. J Nutr 2008; 138: 423S–5S. doi: 10.1093/jn/138.2.423S. 18203915

[13] Burckhardt P The effect of the alkali load of mineral water on bone metabolism: interventional studies. J Nutr 2008; 138: 435S–437S. doi: 10.1093/jn/138.2.435S. 18203918

[14] Wynn E, Raetz E, Burckhardt P The composition of mineral waters sourced from Europe and North America in respect to bone health: composition of mineral water optimal for bone. Br J Nutr 2009; 101: 1195. doi: 10.1017/S0007114508061515. 18775101

[15] Frassetto L, Morris RC, Sebastian A Potassium bicarbonate reduces urinary nitrogen excretion in postmenopausal women. J Clin Endocrinol Metab 1997; 82: 254–9. doi: 10.1210/jcem.82.1.3663. 8989270

[16] Heil DP Acid–base balance and hydration status following consumption of mineral-based alkaline bottled water. J Int Soc Sports Nutr 2010; 7: 29. doi: 10.1186/1550-2783-7-29. 20836884 PMC3161391

[17] König D, Muser K, Dickhuth H-H, Berg A, Deibert P Effect of a supplement rich in alkaline minerals on acid-base balance in humans. Nutr J 2009; 8. doi: 10.1186/1475-2891-8-23. 19515242 PMC2702352

[18] Deutsche Gesellschaft für Ernährung (DGE), Österreichische Gesellschaft für Ernährung (ÖGE), Schweizerische Gesellschaft für Ernährung (SGE) Referenzwerte für die Nährstoffzufuhr. 2. Auflage. Bonn: Neuer Umschau Buchverlag; 2016.

[19] Remer T, Dimitriou T, Manz F Dietary potential renal acid load and renal net acid excretion in healthy, free-living children and adolescents. Am J Clin Nutr 2003; 77: 1255–60. doi: 10.1093/ajcn/77.5.1255. 12716680

[20] Remer T, Manz F Potential renal acid load of foods and its influence on urine pH. J Am Diet Assoc 1995; 95: 791–7. doi: 10.1016/S0002-8223(95)00219-7. 7797810

[21] Remer T, Montenegro-Bethancourt G, Shi L Long-term urine biobanking: storage stability of clinical chemical parameters under moderate freezing conditions without use of preservatives. Clin Biochem 2014; 47: 307–11. doi: 10.1016/j.clinbiochem.2014.09.009. 25239781

[22] Lüthy C, Moser C, Oetliker O. Dreistufige Säure-Basen-Titration im Urin. (Three-phasic acid/base titration in urine.). Med Lab 1977; 30: 174–81. PubMed-ID: . 20563

[23] Hamm LL, Nakhoul N, Hering-Smith KS Acid–base homeostasis. Clin J Am Soc Nephrol 2015; 10: 2232–42. doi: 10.2215/CJN.07400715. 26597304 PMC4670772

[24] Mohammed HM, Abdelatief DA Easy blood gas analysis: implications for nursing. Egypt J Chest Dis Tuberc 2016; 65: 369–76. doi: 10.1016/j.ejcdt.2015.11.009.

[25] Sood P, Paul G, Puri S Interpretation of arterial blood gas. Indian J Crit Care Med 2010; 14: 57. doi: 10.4103/0972-5229.68215. 20859488 PMC2936733

[26] Kellum JA Clinical review: reunification of acid-base physiology. Crit Care Lond Engl 2005; 9: 500–7. doi: 10.1186/cc3789. PMC1297616 16277739

[27] Seifter JL, Chang H-Y Disorders of acid–base balance: new perspectives. Kidney Dis 2016; 2: 170–86. doi: 10.1159/000453028. PMC5260542 28232934

[28] Sirker AA, Rhodes A, Grounds RM, Bennett ED Acid-base physiology: the ‘traditional’ and the ‘modern’ approaches. Anaesthesia 2002; 57: 348–56. doi: 10.1046/j.0003-2409.2001.02447.x. 11939993

[29] Verma AK, Roach P Abnormal laboratory results: the interpretation of arterial blood gases. Aust Prescr 2010; 33: 124–9. doi: 10.18773/austprescr.2010.059.

[30] Awasthi S, Malviya D, Rani R Peripheral venous blood gas analysis: an alternative to arterial blood gas analysis for initial assessment and resuscitation in emergency and intensive care unit patients. Anesth Essays Res 2013; 7: 355. doi: 10.4103/0259-1162.123234. 25885983 PMC4173550

[31] Pitts RF Acid–base regulation by the kidneys. Am J Med 1950; 9: 356–72. doi: 10.1016/0002-9343(50)90431-1. 14771090

[32] Manz F History of nutrition and acid–base physiology. Eur J Nutr 2001; 40: 189–99. doi: 10.1007/s394-001-8346-7. 11842944

[33] Welch AA, Mulligan A, Bingham SA, Khaw K Urine pH is an indicator of dietary acid–base load, fruit and vegetables and meat intakes: results from the European Prospective Investigation into Cancer and Nutrition (EPIC)-Norfolk population study. Br J Nutr 2008; 99 1134–41. doi: 10.1017/S0007114507862350. 18042305

[34] Kessler T, Hesse A Cross-over study of the influence of bicarbonate-rich mineral water on urinary composition in comparison with sodium potassium citrate in healthy male subjects. Br J Nutr. 2000; 84(6): 865–71. 11177203

[35] Siener R, Jahnen A, Hesse A Influence of a mineral water rich in calcium, magnesium and bicarbonate on urine composition and the risk of calcium oxalate crystallization. Eur J Clin Nutr 2004; 58: 270–6. doi: 10.1038/sj.ejcn.1601778. 14749747

[36] Ausman LM, Oliver LM, Goldin BR, Woods MN, Gorbach SL, Dwyer JT Estimated net acid excretion inversely correlates with urine pH in Vegans, lacto-ovovegetarians, and omnivores. J Ren Nutr 2008; 18: 456–65. doi: 10.1053/j.jrn.2008.04.007. 18721741

[37] Max-Rubner-Institut Nationale Verzehrsstudie II. Ergebnisbericht, Teil 2. Karlsruhe, Germany: Max-Rubner-Institut; 2008:307 Available from: https://www.bmel.de/SharedDocs/Downloads/Ernaehrung/NVS_ErgebnisberichtTeil2.pdf?__blob=publicationFile

[38] Lopez HW, Leenhardt F, Remesy C New data on the bioavailability of bread magnesium. Magnes Res 2004; 17: 335–40. PubMed-ID:. 15726909

[39] Greupner T, Schneider I, Hahn A Calcium bioavailability from mineral waters with different mineralization in comparison to milk and a supplement. J Am Coll Nutr 2017; 36: 386–90. doi: 10.1080/07315724.2017.1299651. 28628402

[40] Schneider I, Greupner T, Hahn A Magnesium bioavailability from mineral waters with different mineralization levels in comparison to bread and a supplement. Food Nutr Res 2017; 61: 1384686. doi: 10.1080/16546628.2017.1384686. 29056894 PMC5642192

[41] Naumann J, Sadaghiani C, Alt F, Huber R Effects of sulfate-rich mineral water on functional constipation: adouble-blind, randomized, placebo-controlled study. Complement Med Res 2016; 23: 356–63. doi: 10.1159/000449436. 27924798

[42] Naska A, Lagiou A, Lagiou P Dietary assessment methods in epidemiological research: current state of the art and future prospects. F1000 Res 2017; 6: 926. doi: 10.12688/f1000research.10703.1 PMC5482335 28690835

